# The Inspection of Meat

**Published:** 1906-10-13

**Authors:** 


### The Inspection of Meat.

The subject of meat inspection is very properly prominent in the thoughts of the medical and veterinary professions at the present time. Each profession thinks that the education given to its own members should be sufficient to enable them to perform the duties of a meat inspector in a satisfactory manner, but some of the public think foolishly that a partial veterinary training of six or twelve months is enough to enable a meat inspector to learn his duties. The inspection of meat to be efficient must be carried out at different stages. In the first place, the living animal must be examined to ensure its healthiness, and this can only be carried out by a properly qualified veterinary surgeon. Secondly, the recently killed carcasses should be reported upon whilst the viscera are still available for examination. The meat inspectors in the Central London Markets are said to be greatly hampered in their duties because the meat only comes under their notice when it has been dressed for sale. The liver, spleen, lungs, intestines, and other organs which would naturally be examined for evidence of disease are absent, and it is then only possible to report upon the condition of the flesh. For this, again, the training of the veterinary surgeon is better than that of the ordinary medical man or the unqualified meat inspector who has only had a perfunctory training. These examinations constitute the first line of defence; the second line deals with the wholesomeness of meat exposed for sale, whether in a fresh or preserved state. So far as unsoundness is concerned, the responsibility must always rest with the local authority of any place where the meat is exposed for sale. It is the duty of the local authority to inspect such meat at the shop or other place where it is kept, the observations being continuous so long as the meat remains within the jurisdiction of the authority. A few towns in England have already availed themselves of qualified veterinary surgeons as meat inspectors, and their services have been recognised as satisfactory, both by the butchers and by the public in the districts where they serve. The establishment and maintenance of public slaughter-houses seem at present to be the only way of securing adequate supervision over animals killed for food, and meat so killed should be guaranteed by an official label. In the case of home-killed meat there would be no difficulty as to the acceptance of the label, but in the case of foreign countries this would not be so easy. Dr. Collingridge, the Medical Officer of Health for the City of London, suggests that the experience gained so far by the system of marking for export in Holland and Denmark will be helpful in this question. Only a recognised label sanctioned by the British Government after an inspection officially recognised as satisfactory and in accordance with the standard as regards disease in force in this country at the time would be accepted, and all other meat sent to the clearing depot for inspection before admission to the market. The Government should at once investigate as to the system of inspection adopted in other countries and as to the thoroughness with which such is carried out, and as to the standard in force at the time of such inspection. This can only be done by a prolonged investigation on the spot, and is a matter for which the Government alone should be responsible. Once initiated, if the value of the label were checked from time to time by the meat inspectors, any lapse or change in the standard would be easily discoverable, and a reference could be made back to the Government for further inquiry.

Investigation of a thorough and reliable character is the only guarantee of safety that the public possess in regard to the good or bad quality of food, and they should insist upon it.

In another portion of our issue will be seen how admirably the inspection of food is conducted in New Zealand, a country in which meat inspection is carried out under provisions which have been in existence for many years. This Act insists on inspection at the slaughter-house of the complete animal by skilled and thoroughly competent veterinary surgeons; inspection on removal of the carcass from the slaughter-house; inspection in the cooling-room and in the freezing stores; inspection at the point of shipment, and certification by means of signed label of the high quality and freedom from disease of all exported articles of food. The New Zealand Government have everything in their favour in regard to pasturage, climate, air, and rainfall, and by their methods of food inspection have done a great deal to allay public apprehension and to ensure a supply of wholesome food.

